# Scientific Impact and Its Role in Scientific Reasoning

**DOI:** 10.3390/jintelligence13100129

**Published:** 2025-10-09

**Authors:** Robert J. Sternberg, Alexandra Moravek, Tamara M. Vaz, Riley Mack Schneider

**Affiliations:** 1 Department of Psychology, College of Human Ecology, Cornell University, Ithaca, NY 14853, USA; moraveklexi@gmail.com (A.M.); rms445@cornell.edu (R.M.S.); 2 Grupo PBE Educação, Unijipa University, Rio de Janeiro 76900-079, Brazil; tamaramatiussi.v@gmail.com

**Keywords:** scientific reasoning, scientific impact, drawing conclusions, generating hypotheses, generating experiments, fluid intelligence

## Abstract

We tested 75 participants in a selective university near the East Coast of the United States for their skills in scientific reasoning. We used scientific reasoning assessments for Generating Hypotheses, Generating Experiments, and Drawing Conclusions. To measure scientific reasoning skills, we also used a task involving analyzing scientific impact based on titles of published studies (which were either highly cited or scarcely cited), and another task involving creating what participants believed might be high-impact scientific studies in three subject matter areas. Participants further completed two fluid intelligence tests: Number Series and Letter Sets. They also filled in demographic information, including self-reported SAT/ACT scores and college GPA. (We cannot obtain actual grades at our university because of student-confidentiality issues.) We found that the scientific reasoning tests for Generating Hypotheses, Generating Experiments, and Drawing Conclusions clustered into a single factor, and the task for creating high-impact studies was also factored with these scientific reasoning tests. The two fluid ability tests—Number Series and Letter Sets—clustered into a distinct single factor. The task of analyzing impact seemed to be in between the other tasks, showing characteristics of not only the scientific reasoning tasks but also of the fluid intelligence tasks.

## 1. Introduction

Scientific reasoning, and STEM reasoning, more generally, is the basis for many of the most important innovations of the last century. These innovations include, as a few examples, mRNA vaccines, the discovery of the structure of DNA, the creation of the internet, the development of computers and cell phones that act like miniature computers, the invention of transistors, the development of PET and MRI scanning and the diagnostic medical tests associated with them, the creation of the CRISPR–Cas9 gene-editing tool, the construction of the large Hadron collider at CERN near Geneva, and much more. Scientific reasoning can yield high-impact innovations like these, but only a small percentage of scientific work has the same impact as these innovations. Many scientific studies are scarcely cited, if they are cited at all. How do people reason scientifically, and in particular, how do they produce and assess high-impact scientific work? These are the questions addressed in this article. They also have been addressed, to some extent, in a previous work (Sternberg and Gordeeva 1996).

The rationale for much of this previous work has been that innovative scientific work has its basis in the training that students receive in graduate school; so, one would wish that graduate programs would admit students who excel in scientific reasoning, or at least who can learn to excel. However, it is not clear that the present means of screening and accepting graduate students utilize assessments that measure these important skills. Much of this work has been either largely atheoretical or based on a psychometric theory of general mental ability (Sackett et al. 2020). The work presented here builds on the theory of adaptive intelligence, which is the successor to a theory of successful intelligence (Sternberg 2019; see also Sternberg 1997).

## 2. Theoretical Basis for the Current Research

In the past and current research on scientific reasoning, aspects of the theory of adaptive intelligence (see, e.g., Sternberg 2019) were used as a basis for constructing scientific reasoning tasks. In this theory, so-called “metacomponents” serve as executive processes for scientific reasoning. This reasoning is largely inductive (Nisbett 2016), as opposed to deductive (Guyote and Sternberg 1981). There are no guaranteed answers. These executive processes are as follows:

1. *Recognizing the existence of a scientific problem*. Part of the inductive and creative process in science is recognizing that a potential scientific problem exists, for example, that people seem to be coughing and having runny noses, sore throats, and fevers, but they also have other symptoms that are atypical of colds, flu, or other known illnesses.
2. *Defining the nature of the scientific problem*. A second crucial process is defining exactly what the problem is—what is the nature of the problem that needs to be solved? In the previous example, are the symptoms an atypical form of an existing virus or a new, previously unidentified virus, or are they due to something else altogether?
3. *Representing the scientific problem mentally or otherwise*. This metacomponent is used to mentally sketch out what the problem looks like. In the example, one might do a computer analysis of symptomatology for existing illnesses to determine whether the symptom lists gathered over multiple sufferers match existing patterns. If not, are the symptoms more typical of, say, viruses than other microorganisms?
4. *Deciding the problem is worth pursuing*. One must then decide whether the identified problem is worth one’s time, effort, or other resources. Is it a problem worth solving, or is it better left alone or for someone else to solve? In the example, are enough people showing signs of the unidentified illness? Does it appear to be airborne? Do some or any of the cases seem to be serious?
5. *Allocating resources to the solution of the problem*. If one has decided to pursue the problem, how much time is it worth? How much effort? In the case of the illness, what kinds of material resources will be needed? Is there a need for a budget, and is that budget attainable?
6. *Formulating a strategy to solve the problem*. How is one going to find a solution to the problem? What is the path? In the example, will one perform genetic analysis, epidemiological analysis, microbiological analysis, a combination of these, or something else?
7. *Monitoring solution strategy*. How is the process of finding a solution going? Does it appear to be leading closer to a solution? In the example, is there evidence that the research is leading to the identification of what now appears to be a new virus?
8. *Evaluating the solution after it is reached*. In this process, one evaluates whether the solution fits the problem and answers the questions that were initially raised, plus perhaps some new questions. In the example, the virus behind COVID-19, SARS-CoV-2, is identified, which serves as a basis for combating it.

These processes are all highly relevant to the present study, although they are not measured separately. In designing an experiment, as required in our study, individuals need to (1) recognize the existence of a scientific problem (what is the problem they want to study?), (2) define the scientific problem (how can the problem be operationalized for empirical study?), (3) mentally represent the problem (how will the study be structured to answer the questions it is asking?), (4) decide whether the problem is worth pursuing (is the problem worth my time and effort?), (5) allocate resources (what resources are needed to do the study?), (6) set up a strategy for the achieving the solution (what are the exact steps needed to conduct the empirical study?), (7) monitor problem-solving (is the experiment going as it should be?), and (8) evaluate the solution (how do I analyze the data to understand the results of the study?).

There is no one unique theory that should serve as a basis for understanding scientific reasoning and its components. This is only one theory of many that might account for how scientists think about the problems they confront.

## 3. Antecedent Research on Scientific Reasoning

The work reported here is based primarily on the theory of adaptive intelligence of Sternberg (2019) and its application to scientific reasoning (Sternberg and Sternberg 2017; Sternberg et al. 2017, 2019, 2020). However, many investigators from various fields have sought to understand scientific reasoning. For example, D. Kuhn (2002, 2011) and Kuhn and Dean (2004) have characterized scientific thinking in terms of the formation of a research question or hypothesis, planning and then conducting the scientific investigation, analyzing the results of the investigation, making inferences based on the results, and debating the implications of those results. Dunbar and Klahr (2012) have pointed out that scientific reasoning involves many different and diverse processes of thinking, such as induction, deduction, design of experiments, reasoning causally, concept formation, and testing of hypotheses. Dunbar (1995) found that in real-life labs, scientific thinking is incredibly complex and interactive among scientists. T. S. Kuhn (1970) has pointed out that most scientific thinking is within the paradigms of what he calls “normal science,” or the science of the kind that most scientists do, but some scientists break out of those paradigms and engage in what Kuhn refers to as “revolutionary science.” Koslowski (1996) has pointed out that good scientists do not get stuck in relying dogmatically on existing theories or in just observing and drawing causal conclusions from covariation. Popper (2014) is known for his emphasis on the importance of disconfirmation in science. In contrast, Feyerabend (2010) is famous for his “anything goes” philosophy of science. All these processes are teachable (Sternberg and Spear-Swerling 1999).

Here, we discuss, in somewhat more detail, antecedent research based on two different theories: the theory of general mental ability (GMA) (e.g., Sackett et al. 2020) and the theory of adaptive intelligence (Sternberg 2019).

## 4. Some Research Particularly Based on a Theory of General Mental Ability (GMA)

Tests such as the SAT and GRE are largely tests of general mental ability (Sackett et al. 2020). They are very highly correlated with each other and with IQ (Frey and Detterman 2004; Koenig et al. 2008). Wilson (1979) examined the predictive validity of the Graduate Record Examination (GRE) for first-year grades in psychology. The validity coefficients were .18 for the GRE verbal test, .19 for the GRE quantitative test, and .32 for the GRE analytical test. In a related study, Schneider and Briel (1990) examined the predictive validity of the GRE for grades in the first year of graduate study in psychology. They obtained validity coefficients of .26 for the GRE verbal, .25 for the GRE quantitative test, .24 for the GRE analytical test, and .36 for the psychology achievement (subject matter) test.

Kuncel et al. (2001) used a meta-analysis to study the predictive validity of the GRE in a variety of fields of knowledge. They discovered correlations of .34 for the GRE verbal, .38 for the GRE quantitative, .36 for the GRE analytical, and .45 for the GRE achievement (subject matter) tests. These correlations were double-corrected for both restriction of range (participants in such studies tend to be above average) and attenuation (low reliability). The correlations are, therefore, not the ones obtained in the studies but rather the correlations that would have been obtained, in theory, for an ideal test having perfect reliability that was administered to participants with a full population range of knowledge and skill levels. It is not clear what a “full range” is, because students who would score at the bottom of a distribution are less likely to apply to graduate school (for fear of not being admitted), are less likely to be admitted to graduate school and, later, are less likely to enter the occupation for which the test is a predictor. Kuncel and Hezlett (2007) and Kuncel et al. (2010) also found that standardized tests predict success in graduate school.

Sternberg and Sternberg (2017) argued that a different approach might be more productive, at a theoretical level, for understanding scientific reasoning, and at a practical level, for improving the prediction of graduate-level success in scientific reasoning. Their approach was based on the notion that if one wants to predict future scientific reasoning skills, the best predictor is likely to be present scientific reasoning skills.

## 5. Some Research Based on the Theory of Adaptive Intelligence

Sternberg and Sternberg (2017) based their work on Sternberg’s theories of successful and adaptive intelligence (e.g., Sternberg 1997, 2019). They used five tasks to measure scientific reasoning, as they argued that those tasks represent scientific reasoning as it is actually performed, as opposed to the kind of more general reasoning performed for solving problems on the Graduate Record Examination (GRE):

1. *Hypothesis generation*—formulating plausible alternative hypotheses to explain a phenomenon.
2. *Designing experiments*—producing an experimental design that will test a particular scientific hypothesis.
3. *Drawing conclusions*—interpreting data from an experiment in a way that reflects correct scientific reasoning.
4. *Peer review*—acting as a peer reviewer of a (mock) brief research report.
5. *Editorial decision-making*—acting as an editor who makes decisions about the publishability of a (mock) submission to a journal.

Each of the tasks was scored via a rubric established in advance. In addition, participants received tests of fluid intelligence, namely, Letter Sets and Number Series, and difficult verbal analogies similar to those on the Miller Analogies Test, which measured both crystallized and fluid intelligence. Participants also completed a demographic questionnaire, which included self-reported SAT/ACT scores and grade-point average in college (GPA). There were two previous studies, primarily with undergraduates at a selective university near the East Coast of the United States.

The first three scientific reasoning subscales were moderately to strongly correlated with each other. As discussed in the original work, the results from the other two tasks (peer-reviewing scientific reports and making editorial decisions) were more variable because they proved to be too advanced for the undergraduate participants, who, understandably, were not prepared to be journal reviewers or editors. Reviewing and editing are tasks that are typically not attempted until one starts graduate school, in the case of reviewing, or until one starts working as a faculty member, in the case of journal editing. So, the tasks may well have been at a higher level of novelty than the students were ready to handle in a fully competent way. Thus, the last two tasks (peer review and editorial decision-making) were dropped from the current work, consistent with the earlier research (Sternberg et al. 2017, 2019, 2020) conducted by Sternberg and Sternberg (2017).

Although the scientific reasoning tasks correlated with each other, they were not significantly correlated with the psychometric measures, SAT/ACT scores, or GPA. Some of the scientific reasoning scores showed negative correlations with the SAT/ACT scores. Principal component analysis and principal factor analysis revealed that scientific reasoning scores formed a unified and cohesive factor, as did the psychometric test scores, but they were different factors. The results suggested that scientific reasoning is largely independent of whatever it is that the standardized tests measure.

Sternberg et al. (2019) further investigated the relationships between scientific reasoning and germane constructs. Whereas the earlier study investigated only psychological reasoning, this study investigated reasoning across sciences, including, for example, items in fields such as agriculture and nutrition. The investigators used three scientific reasoning tests, taken from the earlier work: Generating Hypotheses, Designing Experiments, and Drawing Conclusions. (The peer review, editorial decision-making, and Miller Analogies Test types of assessments were not used in this work because, as noted earlier, the college-age participants found them more challenging than expected.) Letter Sets and Number Series were used to measure fluid intelligence, and self-reported results of the SAT/ACT and GPA were also collected.

The key findings of Sternberg et al. (2019) were that the three scientific reasoning tests were intercorrelated and formed a single distinct factor. The scientific reasoning tests did not consistently correlate with the psychometric tests, which were also factored separately. The results were comparable across scientific disciplines. In other words, there was no indication that the particular scientific reasoning skills used in any one field differed from the comparable scientific reasoning skills in any other scientific field.

Another feature of this work was that, in one study, the scientific reasoning items were presented in multiple-choice format. When they were presented in this way, correlations of the measures of scientific reasoning with fluid intelligence increased, suggesting that testing format mattered in terms of what the tests measured. In accordance with our predictions, we could obtain an increase in correlations with fluid intelligence by making test items multiple-choice, but at the expense of ecological validity. In scientific laboratories, problems are rarely, if ever, presented to scientists in multiple-choice format.

In a third study, Sternberg et al. (2017) investigated another part of scientific endeavors, which is the teaching of scientific inquiry. Participants watched sample scientific lectures in which the lecturers were instructed to purposely create lectures that were flawed in prespecified ways. The participants were asked to evaluate and critique what was flawed in the teaching. The measure assessing the quality of evaluation of the teaching was factored with the measures of scientific reasoning, but not with measures of fluid intelligence. Thus, one’s ability to reason about teaching in science appeared to be closely related to one’s ability to reason about research in science.

In recent studies, Sternberg et al. (2020) began investigating the question dealt with in this article. In addition to assessing scientific reasoning, they investigated the ability to evaluate scientific impact and the ability to be scientifically creative and impactful. The Analysis of Scientific Impact measure presented summaries of studies, half of which were low-impact and half of which were high-impact in terms of citation rates. The scientific creativity measure asked participants to design an empirical scientific study on a topic of their choice.

In this work, scientific reasoning formed a distinct factor, similar to the preceding work, with scientific creativity correlating with the scientific reasoning measures and forming a factor with them. The Analysis of Scientific Impact measure either factored with Letter Sets in some studies or formed its own factor in other studies. The evaluation of impact thus appears to measure an ability closer to fluid intelligence, perhaps because the task is quite analytic, i.e., analyzing impact.

The results of this last study were not as predicted. Before the empirical work was conducted, it was (incorrectly) expected that the performance on the task for analyzing the scientific impact would be strongly related to the performance on the scientific reasoning tasks, but this was not the case. In the present research, we asked further questions related to information processing regarding scientific impact measures, including how scientifically rigorous, creative, and practically useful the participants expected the rated study to be. In the previous scientific creativity task, we found that leaving the topic completely open-ended was highly challenging for some participants and also created non-comparability across responses. In this study, we asked participants to design studies on three particular topics with which they were expected to have at least some familiarity.

## 6. Goals of the Current Research

This study had three principal goals:

1. Past research has shown that scientific reasoning yielded a factor distinct from fluid intelligence. Yet, admission to, and fellowship support for, graduate programs in STEM often largely depends on tests of fluid intelligence, not of scientific reasoning. If scientific reasoning is somewhat distinct from fluid intelligence, universities may need to reconsider how they admit students for graduate STEM programs, taking into account scientific reasoning, distinct from fluid intelligence. We sought, therefore, to replicate this result to determine whether the result was robust. Such replication is not only scientifically important but also practically important in a society that relies on conventional admission tests for STEM study, which may measure abilities that are peripheral to those that are most important for success in STEM fields.
2. A previous study found that a measure for evaluating the impact of scientific studies, which was expected to correlate with and load on measures of scientific reasoning, was instead more related to, and factored separately with, measures of fluid intelligence. Because this result was contrary to what was expected, we sought to determine whether the result could be replicated. We believe that this result is important theoretically and practically because the evaluation of the impact of scientific work is crucial to science, whether one is doing research, reviewing research, serving as an editor evaluating research, or serving on a scientific panel to review grant proposals.
3. In past research (Sternberg and Sternberg 2017; Sternberg et al. 2017, 2019, 2020), when creativity was measured, it was of a limited kind, in which the problem was given to participants, who were then asked to design a study on the given topic. Although this procedure created comparability across participants, it did not represent how research is actually conducted (except, perhaps, in corporations and other organizations where scientists are told what problems to solve). Rather, scientists not only solve problems but also define what problems they wish to solve. Thus, it was important to extend the previous research by including a measure where participants would decide for themselves what problem to investigate by designing a study, rather than being told what the problem would be.

This study was not designed as a full construct validation of a particular theory and set of measures. The sample was too small and narrow, and the range of tests was too limited. Rather, it was designed specifically with the purpose of addressing the three issues described immediately above. We believe these questions are theoretically, empirically, and practically consequential, and we sought to address them through our research.

## 7. Method

This study was vetted by the university’s Human Subjects Institutional Review Board and declared exempt, thereby being approved for the collection of data from university students. According to our university’s IRB policy statement, studies are declared exempt through a decision “made by IRB staff based on federally defined criteria like minimal-risk research or secondary data analysis.”

### 7.1. Subjects

This study involved a total of 75 participants. This group included 47 female-identifying participants, 23 male-identifying participants, and 5 participants who declined to answer or identified as “other.” The average age was 20.1, with a range of ages between 18 and 23. The participants consisted of 31 Asians or Asian Americans, 18 European or European Americans, 12 Hispanics or Hispanic Americans, 4 Africans or African Americans, 1 Indigenous American, and 9 participants who declined to respond or answered “other.” All participants were undergraduate students attending a selective university in the Northeast. The participants majored in a variety of fields, but they were taking a behavioral science course in which they would potentially receive course credit for participating in this study.

### 7.2. Materials

All participants completed a series of tasks previously used in or adapted from the materials in Sternberg et al. (2020), with the addition of our new Scientific Impact: Creative assessment. Each of these materials is described below. The scientific reasoning materials used in the previous research (Sternberg 2020; Sternberg and Sternberg 2017; Sternberg et al. 2017, 2019, 2020) have been shown to provide reliable and valid measurements of scientific reasoning. The complete set of materials can be found in Appendix B.

### 7.3. Psychometric Tests

Letter Sets and Number Series were used as our measures of fluid intelligence. These tests had been used in previous studies, in particular, Sternberg et al. (2020), Sternberg and Sternberg (2017), and Sternberg et al. (2017, 2019). These two psychometric tests were shown to have reliability and construct validity in these previous studies. We were limited in our testing by the maximum time limit that the subject pool supports for research utilizing the pool. Fortunately, thousands of studies show that fluid intelligence tests tend to be highly correlated with each other and that it matters little which ones are used (see, e.g., Carroll 1993; Deary 2020; Jensen 1998; Sackett et al. 2020; Spearman 1927).

In each item of the Letter Sets task, participants were presented with five combinations of four letters, for which the participants were asked to find the letter combination that did not match the pattern of the rest of the four sets of letters. Participants were told to look out for a rule that governed all of the combinations, except one, and to find the exception, for example, CDEF, HIJK, QRST, IJKM, and OPQR. Students should have noted that all of the letter combinations, except one, were in alphabetical order; “IJKM” was not in alphabetical order and, therefore, was the “odd man out.” Participants were given 7 min to complete 15 questions.

In each item of the Number Series task, a set of numbers was presented, whereby participants had to utilize pattern recognition abilities. An example of a pattern could be multiples of 2 or each number being repeated twice. Once the participants recognized the pattern, they filled in the number they believed would be presented next in the series, for example, 2, 7, 3, 8, 4, 9,_?__. Participants should recognize that the numbers are increasing by one more than the second-to-last number previously listed, and thus, they should answer 5. In total, 16 Number Series were presented, and an allotted time of 7 min was given to answer the questions.

### 7.4. Scientific Impact: Analytical Assessment

Participants were presented with 20 titles of research articles, each of which had either more than 2000 citations, denoted as “high-impact,” or fewer than 15 citations, denoted as “low-impact” (see Sternberg et al. 2020 for the previous use of such a task). In the earlier work in the related series of studies (e.g., Sternberg and Sternberg 2017), full abstracts were given, as well as titles, but it was later found (e.g., Sternberg et al. 2020) that the results were quite similar with just the titles and without the abstracts. We suggest that this similarity results from participants being able to infer from the title the importance of the problem being studied. “Cuter” or more clever titles were rated as more creative but not necessarily as more scientifically impactful.

Based on only the title of the research article, participants were asked to characterize the article as high- or low-impact. The articles were sourced from PubMed, Web of Science, and Google Scholar databases, where the number of citations was listed. The participants were told before they started the task that half of the items would correspond to a high-impact study while the other half would correspond to a low-impact study. Articles were chosen from a variety of topics, including psychology, medicine, nutrition, environmental science, anthropology, and ethnic studies.

Note that articles were from fields and dates that were varied on purpose. For example, the scientific fields included psychology, sociology, medicine, biology, anthropology, engineering, and nutritional science. The dates of the studies ranged from 1978 to 2013 (1 from 1978, 1 from 1998, 1 from 1999, 2 from 2000, 1 from 2002, 2 from 2003, 1 from 2010, 3 from 2011, 3 from 2012, and 5 from 2013). Although different fields have different rates of citation, all the fields chosen involved a large number of scientific contributors from all over the world and thus the potential for a high rate of citations; moreover, at the time this study was conducted, the articles had been publicly available long enough to achieve high citation rates. In particular, some of the older articles were not highly cited, while some of the more recent ones were.

Participants were also asked to rank, on a scale of 1 to 3, how (a) creative, (b) scientifically rigorous, and (c) practically useful they found each study to be and how (d) confident they were in their impact rating. Here is an example of this type of question:

“The ‘what’ and ‘why’ of goal pursuits: Human needs and the self-determination of behavior”

- Do you believe this study to be high-impact—cited many times—or low-impact—cited very few times (H or L)?
- How confident are you in your rating (3 [high confidence], 2 [medium confidence], or 1 [low confidence])?
- How creative do you believe this work to be (3 [highly creative], 2 [somewhat creative, 1 [slightly creative])?
- How scientifically rigorous do you believe this work to be (3 [highly rigorous], 2 [somewhat rigorous], 1 [slightly rigorous])?
- How practically useful do you believe this work to be (3 [highly practically useful], 2 [somewhat practically useful], 1 [slightly practically useful])?”

### 7.5. Scientific Impact: Creative Assessment

In Sternberg et al. (2020), the measure of Scientific Impact—the same assessment that was just described—showed mixed results in principal-component analysis and principal-factor analysis. In the three studies that were performed, the Scientific Impact measure did not factor clearly with Scientific Reasoning. Instead, Scientific Impact was sometimes factored with fluid intelligence tests and sometimes by itself. The idea was that the task may be designed to test the Analysis of Impact, which is an important skill in the scientific process, but perhaps less so than the ability to come up with one’s own impactful research ideas. In that case, the Scientific Impact task would be more analytical than creative.

The Scientific Creativity task in Sternberg et al. (2020) asked participants to design a scientific study to test an important question about a specific topic that was presented to them. This task was correlated and factored with the Scientific Reasoning tests; thus, we used an adapted version of this task in our study to further evaluate one’s ability to grasp scientific impact. We defined impact for participants below:

“Studies that are more impactful on society sometimes are designated as ‘high-impact’ studies. Impact can be measured via the number of citations of a given published work. Scientific works that are cited very few times tend to have a lower impact on science and society. These works can be considered ‘low impact.’”

Then, we asked the participants to design, specifically, a high-impact study about a general topic or trend. Due to the creative nature of the prompt, we expected that this new, creative evaluation of scientific impact would factor with the Scientific Reasoning measures and could thus be included as a measure of scientific thinking abilities. The creativity task differed from that in the previous work in three major respects: (1) the participants were explicitly asked to design a high-impact study (consistent with our emphasis in this study on scientific impact); (2) the participants were given topics on which to write, rather than being left to choose their own topic, ensuring greater comparability across answers in measuring individual differences; and (3) the participants were asked to design three studies, rather than just one, to ensure greater generalization through the three topics (rather than one). Here are the further tasks:

**Task 1:**

Imagine you were given a task to design a high-impact study that would help understand the high rates of depression among adolescents. Your response should be approximately 200–300 words describing:

(a) the idea for the study,
(b) the goal of the study,
(c) your hypothesis or hypotheses regarding the outcomes of the study,
(d) what you will be measuring, and
(e) how the study would be performed.

**Task 2:**

In the context of the COVID-19 pandemic, design a high-impact study, as defined above, that you believe would be important in regard to the use of face masks. Your response should be approximately 200–300 words describing:

(a) the idea for the study,
(b) the goal of the study,
(c) your hypothesis or hypotheses regarding the outcomes of the study,
(d) what you will be measuring, and
(e) how the study would be performed.

**Task 3:**

Given the current climate crisis the world is facing today, formulate a high-impact study you would perform to express to current policymakers the urgent need for a policy change. Examples of issues regarding climate include carbon emissions, pollution, overfishing, fertilizer run-off, etc. Your response should be approximately 200–300 words describing:

(a) the idea for the study,
(b) the goal of the study,
(c) your hypothesis or hypotheses regarding the outcomes of the study,
(d) what you will be measuring, and
(e) how the study would be performed.

We scored responses based on a 15-point scale, in which each section (a, b, c, d, and e) was graded individually on a scale of 0 to 3. Three raters were used to grade the responses. After each rater had graded individually, discrepancies between raters were discussed, and a consensus was determined. The guidelines for each part of the question are shown below:

0—no response.
1—responded, but the response was weak and not novel or useful.
2—developed a response that was either practically useful or novel, but not both.
3—developed a strong, novel, and practically useful response.

### 7.6. Scientific Reasoning

Previous research by Sternberg et al. (2020), Sternberg and Sternberg (2017), and Sternberg et al. (2017, 2019) used 3 assessments for scientific reasoning: Generating Alternative Hypotheses, Generating Experiments, and Drawing Conclusions. We used these 3 types of questions, taken from the previous work, for our own measures. Examples of the questions are described below.

I. **Generating Hypotheses**

Participants were presented with a scenario and a hypothesis that would explain a behavior in a situation and were then asked to come up with other alternative explanations for the behavior described in the situation. An example of a question we used is as follows:

“Marie is interested in child development. One day, she notices that whenever Laura’s nanny comes in to pick up Laura from nursery school, Laura starts to cry. Marie reflects upon how sad it is that Laura has a poor relationship with her nanny.

*What are some alternative hypotheses regarding why Laura starts to cry when she is picked up from nursery school by the nanny?”*

Participants provided as many alternative hypotheses as they wished. Alternative hypotheses were graded as plausible or implausible. Responses that were plausible were given one point, while responses that were not plausible were given zero points; thus, participants were awarded a point for every plausible hypothesis they provided.

II. **Generating Experiments**

For this next section, participants were presented with two vignettes and an accompanying hypothesis for each. Then, participants were asked to design an experiment to test the hypothesis and how they would perform the experiment. Here is an example:

“John hypothesizes that his brother’s playing of violent video games has increased his brother’s aggressive behavior. John is not sure, however, whether playing violent video games really increases aggression.”

*Please suggest an experimental design to test John’s hypothesis and describe the experiment in some detail. Assume you have the resources you need to be able to do the experiment (e.g., access to violent video games, subjects, sufficient funds to pay subjects, etc.).*

The quality of each answer was graded on a scale of 1 to 5:

1 = unsatisfactory.
2 = minimally satisfactory; answers the question, but the response is weak.
3 = highly satisfactory; goes a step beyond the minimum.
4 = good; the answer is well beyond satisfactory.
5 = outstanding answer.
0 = missing value.

A value of 2 is roughly what we expected as the mean score on each question for this section; we expected a score of 5 to be quite rare. While scoring, we considered whether the proposed study (a) included a control group, (b) tested what it was supposed to test, (c) included random assignment of subjects, etc. The inclusion of these factors contributed to a higher score.

III. **Drawing Conclusions**

The last section pertaining to Scientific Reasoning involved Drawing Conclusions. Participants were presented with two scenarios that described an experiment to test a specific hypothesis. The participants had to consider the design of the experiment and communicate any flaws in the conclusions drawn from the data. An example of a question we used was as follows:

“Bill was interested in how well a new program for improving mathematical performance worked. He gave 200 students a pretest on their mathematical knowledge and skills. He then administered the new program to them. After administering the program, he gave the same 200 students a posttest that was equal in difficulty and in all relevant ways comparable to the pretest. He found that students improved significantly in performance from pretest to posttest. He concluded that the program for improving mathematical performance was effective.”

*Is this conclusion correct? Why or why not?*

Responses were graded according to the same scale of 1 to 5 that was used to grade the experimental design section, otherwise referred to as Generating Experiments.

### 7.7. Demographic Questionnaire

The last section of this study gathered demographic information on the participants. The information collected included the gender, age, ethnicity, major, undergraduate grade-point average (GPA), self-reported SAT and/or ACT scores, and amount of experience in research. We had confidence in the self-reported SAT/ACT scores because previous research found that these self-reported scores tend to show high validity (Cole and Gonyea 2010).

### 7.8. Design

The study encompassed a within-subject design, in which all participants completed all tasks. We were interested in discovering the latent principal components and common factors that would underlie our new “Scientific Impact: Creative” task and other observable variables. More specifically, we investigated whether our new assessment, “Scientific Impact: Creative,” would factor with measures of Scientific Reasoning. We predicted a positive relationship.

### 7.9. Procedure

This study was conducted at a selective university in the Northeastern United States. The materials were arranged in the following way: (1) informed consent form, (2) Letter Sets, (3) Number Series, (4) Scientific Impact: Creative, (5) Scientific Impact: Analytical, (6) Generating Hypotheses, (7) Generating Experiments, (8) Drawing Conclusions, (9) demographic questionnaire, and (10) debriefing information. The only timed tasks were Letter Sets and Number Series, for which participants were allocated 7 min to complete each test. This study was conducted online via a Qualtrics survey, and access was available through Sona Systems. Participants were granted SONA credit for participation in this study.

## 8. Results

This study aimed to extend prior research on the relationships among scientific creativity, the evaluation of scientific impact, scientific reasoning, and general intelligence (see Sternberg et al. 2020). Unlike earlier studies, the current work focused specifically on the creative production of impactful ideas and examined how the Scientific Impact measures align with other dimensions of scientific thinking.

The results are organized into three key sections: descriptive statistics, correlations, and factor analyses.

All assessments were scored using either objective answer keys (e.g., Letter Sets and Number Series) or detailed rubrics (e.g., Scientific Impact and Scientific Reasoning). This approach ensured consistency and rigor in evaluating cognitive and creative performance across measures.

### 8.1. Descriptive Statistics

Table 1 summarizes the descriptive statistics for the measures used in this study. These measures include self-reports of standardized admissions tests (self-reported SAT scores and ACT scores that were converted to SAT scores), self-reported undergraduate grade-point average (GPA), psychometric assessments (Letter Sets and Number Series), and self-reported amount of experience in research.

Additionally, Table 1 presents the statistics for the tools used to evaluate Scientific Impact from both creative and analytical perspectives, as well as the measures assessing Scientific Reasoning across the three tasks: Generating Hypotheses, Generating Experiments, and Drawing Conclusions.

Participants rated Scientific Impact: Analytical ratings by categorizing each study as either high- or low-impact. Scores were based on whether participants provided correct or incorrect responses, with a chance level set at 50%. As demonstrated in Sternberg et al. (2020), the test items were found to be meaningful and the task was of appropriate difficulty, with participants performing at above-chance levels.

The average GPA of participants was 3.59 (on a 4.33 scale, where 4.33 corresponds to an A+). Regarding standardized cognitive measures, the mean self-reported SAT scores were 723.1 for Reading and 748.8 for Math, reflecting academic abilities consistent with students from highly selective institutions. However, notable variation was observed, with standard deviations of 68.47 for Reading and 67.88 for Math. For reference, according to the U.S. Department of Education (2023), SAT standard deviations at the population level typically range around 114, although variability exists across states, with some reporting figures closer to the 90s.

ACT scores were converted to the equivalent of their SAT scores using the most recent ACT/SAT Concordance Tables (ACT and College Board 2018) for consistency. Moreover, the number of participants providing SAT/ACT scores was 10 fewer than those who completed the other tasks summarized in Table 1. Despite this variation, the self-reported SAT/ACT scores indicate that this study consisted of a strong sample, typical of students at highly selective institutions.

Given the limited sample size, analyses by gender and ethnicity were not conducted.

### 8.2. Inter-Rater Reliabilities

Inter-rater reliabilities for the various measures created by the authors (computed by the intra-class correlation method) were Generating Hypotheses: .995, Generating Experiments: .885, Drawing Conclusions: .945, Scientific Impact—Creative Question 1: .933, Scientific Impact—Creative Question 2: .940, and Scientific Impact—Creative Question 3: .908.

### 8.3. Correlations

Appendix A (Table A1) presents the intercorrelations among the various measures included in this study. Only statistically significant correlations at the *p* < 0.05 and *p* < 0.01 levels are discussed. It is important to note that conclusions drawn from nonsignificant findings are limited by the statistical power of the correlational tests.

There are several key results:

First, the correlation between the Scientific Impact: Creative scores and the Scientific Impact: Analytical scores was significant (*r* = 0.33, *p* < 0.01), supporting the notion that these two constructs are indeed related. This correlation is consistent with the nature of the tasks, as the two tasks (analytical and creative) both aim to measure aspects of understanding scientific impact.

Second, as expected, our three Scientific Reasoning measures were significantly intercorrelated with each other. Generating Hypotheses significantly correlated with Generating Experiments (r = 0.39, *p* < 0.01). Drawing Conclusions also significantly correlated with Generating Hypotheses (r = 0.36, *p* < 0.01). Finally, Generating Experiments correlated significantly with Drawing Conclusions (r = 0.51, *p* < 0.01). These data suggest coherence in the reasoning abilities evaluated among these assessments, consistent with the past related studies.

Third, Scientific Impact: Creative scores significantly correlated with all Scientific Reasoning measures (*r* = 0.24, *p* < 0.05; *r* = 0.53, *p* < 0.01; *r* = 0.41, *p* < 0.01), which is consistent with the similarity in task demands; both required participants to generate experimental designs, although the Scientific Impact: Creative measure involved more detailed formulating of the problem. This set of results also aligns with the expectation that creativity plays a role in designing studies and interpreting results.

Fourth, among the Scientific Reasoning tasks, only Generating Experiments was significantly correlated with Scientific Impact: Analytical (*r* = 0.31, *p* < 0.01), suggesting a shared emphasis on experimental logic.

Fifth, the conventional ability measures, i.e., Letter Sets and Number Series, were significantly correlated with each other (*r* = 0.44, *p* < 0.01), as expected, given their shared measurement of fluid intelligence. However, they generally did not correlate with Scientific Reasoning or Scientific Impact measures. Notable exceptions included Letter Sets correlating with Scientific Impact: Analytical (*r* = 0.32, *p* < 0.01), suggesting that fluid intelligence may be more closely linked to individuals’ ability to identify the impact of studies than their ability to generate creative studies, as found in the previous studies. Additionally, Number Series correlated significantly with Scientific Reasoning: Generating Experiments (*r* = 0.27, *p* < 0.05).

Sixth, Letter Sets were negatively correlated with Research Experience (*r* = −0.35, *p* < 0.01); also, Number Series was negatively correlated with the Number of Scientific Articles Read (*r* = −0.24, *p* < 0.05).

Seventh, SAT Reading and SAT Math scores were significantly correlated (*r* = 0.32, *p* < 0.01). Although consistent with previous findings, surprisingly, SAT scores and Undergraduate GPA were not significantly correlated with Number Series or Letter Sets, but SAT Math was correlated with Number Series (*r* = 0.31, *p* < 0.05). Additionally, SAT Reading and Math scores were not correlated significantly with Scientific Impact or Scientific Reasoning measures, suggesting that, at least in our sample, SAT performance does not predict the scientific skills assessed in this study.

Eighth, the Number Of Scientific Articles Read was positively correlated with Scientific Impact: Creative (*r* = 0.24, *p* < 0.05), Scientific Reasoning: Generating Experiments (*r* = 0.24, *p* < 0.05), Scientific Reasoning: Drawing Conclusions (*r* = 0.31, *p* < 0.01), Scientific Impact: Analytical (*r* = 0.27, *p* < 0.05), Undergrad GPA (*r* = 0.24, *p* < 0.05), and Research Experience (*r* = 0.26, *p* < 0.05), suggesting that exposure to the scientific literature may facilitate more creative thinking in research contexts.

Finally, Scientific Reasoning: Drawing Conclusions was positively associated with Research Experience (*r* = 0.36, *p* < 0.01) and Research Methods Course Taken (*r* = 0.31, *p* < 0.01). These associations indicate that practical and methodological training are associated with individuals’ ability to interpret scientific data and draw reasonable conclusions.

Each correlation was also evaluated for nonlinear relations. However, graphical analysis indicated that the relationships were largely linear.

As shown in Table 2, participants’ ratings of article attributes, provided in the Scientific Impact: Analytical task, revealed three notable correlations: ratings of Scientific Creativity correlated significantly with both scientific rigor (r = 0.48, *p* < 0.01) and practical usefulness (r = 0.42, *p* < 0.01), and ratings of scientific rigor and practical usefulness were strongly correlated (r = 0.55, *p* < 0.01). These findings suggest that participants perceived more creative articles as being both rigorous and practically useful.

### 8.4. Paired Sample t-Tests

To examine whether significant differences existed between high-impact and low-impact studies in the Scientific Impact: Analytical assessment, we conducted a paired sample t-test. This analysis compared ratings on three dimensions: creativity, practical usefulness, and scientific rigor. Although we formulated hypotheses, suggesting one-sided (tailed) *t*-tests, we also present the two-sided *t*-tests as a matter of conservatism in presentation.

We hypothesized that high-impact studies would receive higher ratings for all three dimensions: practical usefulness, scientific rigor, and creativity. The results supported our expectations for practical usefulness and scientific rigor but did not reveal significant differences for creativity.

1. **Creativity**: Low-impact studies had slightly higher creativity ratings (*M* = 22.56, *SD* = 3.31) compared to high-impact studies (*M* = 21.87, *SD* = 3.71). However, this difference was not statistically significant: *t* (74) = −1.56, one-sided *p* = .061, two-sided *p* = .123.
2. **Practical Usefulness**: High-impact studies were rated significantly higher in practical usefulness (*M* = 25.35, *SD* = 2.66) than low-impact studies (*M* = 20.16, *SD* = 3.13), with *t* (74) = 12.24 and *p* < .001.
3. **Scientific Rigor**: Similarly, high-impact studies scored significantly higher in scientific rigor (*M* = 22.99, *SD* = 3.17) compared to low-impact studies (*M* = 20.47, *SD* = 2.95), with *t* (74) = 7.55 and *p* < .001.

High-impact studies demonstrated clear advantages in practical usefulness and scientific rigor, consistent with our hypotheses. This was the same trend as observed in study 3 of Sternberg et al. (2020). Creativity ratings did not differ as a function of impact. This result may be because the less impactful studies tended to have catchier titles and titles that were specific to narrow or specific contexts.

Table 3 and Table 4 summarize the results.

### 8.5. Factor Analyses

Both principal component analysis and common factor analysis were performed. The number of factors was determined jointly by considering eigenvalues (greater than 1), scree plot analysis, consistency with past results with the battery of tests, and the psychological interpretability of factors.

The principal component analysis (PCA) results, as shown in Table 5, identified three distinct components based on a Varimax rotation. Component 1 loaded Scientific Reasoning tasks, including Generating Hypotheses (0.72), Generating Experiments (0.76), and Drawing Conclusions (0.79). Additionally, our new task, Scientific Impact: Creative, loaded on Component 1 (0.61) as well. This result pattern suggests that our new measure of participants’ ability to creatively generate impactful research ideas measures largely the same skills as our Scientific Reasoning tasks.

In contrast, Component 2 demonstrated associations with fluid intelligence, as evidenced by strong loadings for the Letter Sets (0.82) and Number Series (0.82) tasks. Neither measure of scientific impact loaded strongly on this component.

Component 3 included notable loadings for Scientific Impact: Analytical (0.81) and Scientific Impact: Creative (0.54) tasks. In Sternberg et al. (2020), the Scientific Impact: Analytical task was factored with fluid intelligence tests or by itself in the three studies that were conducted; thus, these results are consistent with previous research. However, these data also highlight a connection between the skills used to recognize impactful ideas and creatively generate them yourself. Thus, our novel Scientific Impact: Creative task utilizes both scientific reasoning and analytical intelligence skills.

In addition to the principal component analysis (PCA) (Table 5), we used a principal axis factor analysis with a Varimax rotation, as shown in Table 6. These results showed a similar trend to the PCA. Factor 1 loaded Scientific Reasoning measures: Generating Hypotheses, Generating Experiments, and Drawing Conclusions and less strongly loaded Scientific Impact: Creative. Factor 2 consisted of Scientific Impact: Creative, Generating Experiments, and Scientific Impact: Analytical. Lastly, Letter Sets and Number Series comprised Factor 3.

Both a principal component analysis and a principal axis factor analysis were also performed with an Oblimin rotation to consider oblique factors. The results were largely the same, with a few discrepancies. For the oblique-rotated principal component analysis, the correlation between Component 1 and Component 2 was 0.295, that between Component 1 and Component 3 was −0.031, and that between Component 2 and Component 3 was −0.066. For the oblique-rotated principal factor analysis, the correlation between Factor 1 and Factor 2 was 0.436, that between Factor 1 and Factor 3 was 0.151, and that between Factor 2 and Factor 3 was −0.039. A notable difference was that Generating Hypotheses factored by itself in Component 3 of the PCA with an Oblimin rotation, whereas Component 3 of the PCA with a Varimax rotation loaded Scientific Impact: Creative and Scientific Impact: Analytical. Furthermore, Component 1 of the principal axis factor analysis loaded for all tasks, except Letter Sets and Number Series (the fluid intelligence tests), including Scientific Impact: Creative, Generating Hypotheses, Generating Experiments, Drawing Conclusions, and Scientific Impact: Analytical. Then, similar to the PCA, Component 3 of the PFA with an Oblimin rotation loaded Generating Hypotheses by itself. This difference may be because Generating Hypotheses introduces a fluency component—one must think of as many alternative hypotheses as possible; such a fluency component was not required in the other Scientific Reasoning tests. 

## 9. Discussion

The purpose of this study was, in part, to conceptually replicate past results on scientific reasoning and thereby test for their durability, as well as to further explore scientific creativity with relation to high-impact work. The tasks were based on the metacomponents of the theory of adaptive intelligence (Sternberg 2019).

The Generating Hypotheses task requires metacomponents that were described above. In particular, generating alternative hypotheses in science requires scientific thinkers to consider alternative definitions of problems and to consider which of those alternative definitions would be worth considering when designing research to counter alternative explanations of a phenomenon, i.e., not only how one can obtain support for one’s preferred hypothesis but also provide evidence against alternative competing hypotheses.

In Generating Experiments, one must recognize a problem, define it, represent it, decide whether it is worth studying, represent it, in part, through hypotheses about outcomes, set up a set of experimental operations to test one’s hypotheses, monitor the solution, and then evaluate the results.

In Drawing Conclusions, one must use, in particular, the metacomponent of evaluating one’s conclusions (the last metacomponent described earlier). What conclusions can be validly drawn from the research that has been performed? One must also ensure that the conclusion is relevant to the problem as it was originally defined during the metacomponential process of problem definition.

We believe that the results are quite illuminating. The results of the rotated principal component analysis and principal factor analysis were similar, but the solution for the rotated principal component analysis was clearer. The three scientific reasoning assessments, similar to all previous research on these measures, clustered together into a single factor. The elaborated scientific creativity measure, used for the first time in this study, also loaded on this factor (more strongly in the principal component analysis than in the principal factor analysis). Similar to the previous study (Sternberg et al. 2020), the measure of Analysis of Scientific Impact did not load on this factor. A second factor clearly measured fluid intelligence with both Letter Sets and Number Series. In both the principal component analysis and principal factor analysis, the Analysis of Scientific Impact did not show meaningful loadings with the factor for Letter Sets and Number Series. In the principal component analysis and factor analysis, the two measures related to scientific impact, i.e., an analytic one and a creative one, both appear to have loaded on a factor, Factor 3 and Factor 2, respectively. In the principal factor analysis, Factor 1 showed high loadings for the three scientific reasoning measures: .61 for Generating Hypotheses, .57 for Generating Experiments, and .56 for Drawing Conclusions; Scientific Impact: Creative loaded only .33. Factor 2 of the principal factor analysis comprised Scientific Impact: Creative at .61, Generating Experiments at .56, and Analysis of Impact at 0.49. Factor 3 of the PFA showed loadings of Letter Sets and Number Series of .79 and .51, respectively, whereas Scientific Impact: Analytical loaded more weakly at 0.32.

The results suggest, therefore, that fluid intelligence forms a clear factor, scientific reasoning forms a clear factor (including the two creative measures), and analysis and creative work, with regard to scientific impact, seem to be somewhere in the middle, which was also the case in Sternberg et al. (2020). The previous results (Sternberg and Sternberg 2017; Sternberg et al. 2017, 2019, 2020) thus seem to have been replicated and extended.

The results suggest that measures of scientific reasoning, based on the metacomponents of the theory of adaptive intelligence (Sternberg 2019), can be useful in evaluating individuals’ present and, perhaps, future scientific reasoning skills. However, they are not the only measures that can be useful in some way. Although the atheoretical studies described above were performed, we believe that, primarily as validations of tests—such as the SAT or the GRE—they can be viewed as based on a theory of general intelligence—*g* (e.g., Carroll 1993; McGrew 2005; Spearman 1927)—and as illustrations of the power of general mental ability (Sackett et al. 2020). The studies show the predictive validity of GMA-based measures for predicting grades and other academic measures, and other studies have shown that GMA-based measures also predict certain successful outcomes in scientific careers (e.g., Bernstein et al. 2019; McCabe et al. 2020). The GMA measures are compatible with measures based on the theory of adaptive intelligence. Success in science, as in anything else, is multi-faceted.

In terms of the questions posed initially, we found the answers we sought. First, we replicated the finding whereby scientific reasoning and fluid intelligence measures segregated into distinct factors. Second, we found that the Analysis of Scientific Impact measure clustered factorially with the Scientific Impact: Creative measure, as well as with Designing Experiments. Thus, in this study, we obtained two factors distinct from the fluid intelligence measures. Third, we found that the scientific creativity measures—whether the problems were given to participants or whether they were original to the participants—clustered together.

This study, like all studies, has weaknesses that need to be addressed in future research.

First, the number of participants was small, and the participants were all students at a selective university in the East of the United States. However, this selectivity may be less of a problem than it appears to be. Students who study STEM and get degrees in it, which can be used for professional purposes, are well above the population average. For example, according to the National Center for Education Statistics, the average SAT scores (which are each scaled to have a mean of 500 for the standardization sample) for majors in the physical sciences are 591 and 595; in engineering, they are 560 and 580, and in computer and information sciences, they are 568 and 575 (NCES 2017). These scores, however, are nationwide and include individuals who have little desire to, or perhaps an aptitude for, actually pursuing a career in STEM. Those who pursue a career in STEM are likely to be considerably more talented than the average STEM student. Also, the individuals who go on to make major contributions often have much higher SAT scores (McCabe et al. 2020). Thus, the above-average sample we investigated is perhaps not highly atypical of those who go on to have distinguished, or even successful, STEM careers. However, our results may not be generalizable to populations that cover a wider range of GMA levels.

Second, the scientific reasoning measures are not standardized, and it is not clear that they will be, at least in the near future. We do not have the wherewithal in our university setting to standardize tests.

Third, we cannot say that these results would hold up cross-culturally, or even for students of different subcultures within the United States. Although we have no particular reason to expect any differences across cultures or subcultures, without doing the research, we cannot say for sure what the results would be elsewhere. Intelligence functions somewhat differently across cultures (Sternberg and Grigorenko 2006; Sternberg et al. 2022).

Fourth, there was no external validation of success in scientific careers, which is what our measures are ultimately designed to predict. The measures are not designed, in particular, to predict results in STEM courses, in which success tends to rely more on fluid intelligence because a large part of such courses is learning course content and solving canned problems, neither of which requires much or, possibly, any scientific creativity. We simply do not have the resources to conduct the very long-term follow-ups that would be required to keep track of the participants (who were anonymous, in any case, and thus could not be followed over time) and then to test those who chose to follow STEM careers.

Fifth, although this study analyzes scientific reasoning and various aspects of such reasoning, it does not elicit basic information processing components of such reasoning (see, e.g., Sternberg 1985, 1994). Such an analysis should perhaps be forthcoming.

Sixth, we used citations as a measure of scientific impact. We make no claim that citations are a comprehensive measure of such impact (Dougherty and Horne 2022), but we needed a reasonable measure that captured at least some, although certainly not all, major features of scientific impact, and it is widely agreed upon that citations serve that purpose (Hirsch 2005, 2007, 2010; Margolis 1967; Radicchi et al. 2008; Sternberg 2016).

Seventh, we found some puzzling results. Letter Sets were negatively correlated with Research Experience, and Number Series was negatively correlated with the Number of Scientific Articles Read (*r* = −0.24, *p* < 0.05). We do not know why these correlations were negative. One could speculate that students who do not perform as well on standardized tests are more likely to obtain more research experience to compensate for lower scores in future applications for graduate school. In general, we found that scientific skills were not substantially correlated with GMA skills, and it may be that those who have devoted more attention to their scientific development are those who have focused on developing these specific scientific reasoning skills rather than general abstract reasoning skills. However, such interpretations are purely speculative.

Finally, the results of the principal component analysis and principal factor analysis were similar, but not identical. The differences might or might not be replicated. We simply do not know. However, the results do suggest that an understanding of scientific impact can be explored in terms of both the creative and analytical processes involved. These processes are different, and hopefully, their relationships will become clearer over time.

## Figures and Tables

**Table 1:** Descriptive statistics.

Assessment	Mean	SD	Skewness	Kurtosis	N
Letter Sets	10.09	3.07	−0.92	0.45	75
Number Series	12.29	2.75	−0.90	0.55	75
Scientific Impact: Creative	20.93	8.25	−0.05	−0.60	75
Scientific Impact: Analytical	13.93	2.50	−0.40	−0.02	75
Scientific Reasoning: Generating Hypotheses	3.65	3.01	1.79	2.68	75
Scientific Reasoning: Generating Experiments	4.36	1.66	0.70	1.44	75
Scientific Reasoning: Drawing Conclusions	4.07	1.97	0.27	0.19	75
GPA	3.59	0.44	−1.03	0.41	71
Research Experience	1.24	0.69	−0.36	−0.87	65
Lab Courses Taken	1.96	2.05	0.99	0.34	69
Research Methods Course Taken	1.47	0.50	0.11	−2.04	72
Number of Scientific Articles Read	5.30	8.36	4.41	26.09	71
SAT Reading	723.10	68.47	−2.09	7.55	65
SAT Math	748.80	67.88	−1.58	2.09	65

**Table 2:** Correlations (*N* = 75).

		Creative	Scientifically Rigorous	Practically Useful
Creative	Pearson Correlation	1.00	0.48 **	0.42 **
Scientifically Rigorous	Pearson Correlation	0.48 **	1.00	0.55 **
Practically Useful	Pearson Correlation	0.42 **	0.55 **	1.00

** Correlation is significant at the 0.01 level (2-tailed).

**Table 3:** Statistics for paired samples (N = 75).

		Mean	Std. Deviation	Std. Error Mean
Pair 1	High-Impact Creativity	21.87	3.71	0.43
	Low-Impact Creativity	22.56	3.31	0.38
Pair 2	Highly Practically Useful	25.35	2.66	0.31
	Slightly Practically Useful	20.16	3.13	0.36
Pair 3	Highly Scientifically Rigorous	22.99	3.17	0.37
	Slightly Scientifically Rigorous	20.47	2.95	0.34

**Table 4:** Paired sample test (high impact–low impact).

		Paired Differences					t	df	Significance	
		Mean	Std. Deviation	Std. Error Mean	95% Confidence Interval of the Difference				One-Sided p	Two-Sided p
					Lower	Upper				
Pair 1	Creativity	−0.69	3.84	0.44	−1.58	0.19	−1.56	74.00	0.061	0.123
Pair 2	Practically Useful	5.19	3.67	0.42	4.34	6.03	12.24	74.00	0.000	0.000
Pair 3	Scientifically Rigorous	2.52	2.89	0.33	1.85	3.19	7.55	74.00	0.000	0.000

**Table 5:** Rotated component matrix.

	Component		
	1	2	3
Letter Sets	0.08	0.82	0.19
Number Series	0.10	0.82	0.07
Scientific Impact: Creative	0.61	0.02	0.54
Scientific Reasoning: Generating Hypotheses	0.72	0.30	−0.35
Scientific Reasoning: Generating Experiments	0.76	0.14	0.32
Scientific Reasoning: Drawing Conclusions	0.79	−0.003	0.13
Scientific Impact: Analytical	0.10	0.27	0.81
Extraction Method: Principal Component Analysis. Rotation Method: Varimax with Kaiser Normalization. The three components accounted for 69% of the variance in the data.			
a. Rotation converged in 7 iterations.			

**Table 6:** Rotated factor matrix.

	Factor		
	1	2	3
Letter Sets	0.09	0.12	0.79
Number Series	0.14	0.14	0.51
Scientific Impact: Creative	0.33	0.61	0.10
Scientific Reasoning: Generating Hypotheses	0.61	0.03	0.18
Scientific Reasoning: Generating Experiments	0.57	0.56	0.12
Scientific Reasoning: Drawing Conclusions	0.56	0.35	0.04
Scientific Impact: Analytical	0.00	0.49	0.32
Extraction Method: Principal Axis Factoring. Rotation Method: Varimax with Kaiser Normalization.			
a. Rotation converged in 9 iterations. The three factors accounted for 69% of the variance in the data.			

## Data Availability

Data are available from the senior author upon request.

## Appendix A

**Table A1:** Complete Intercorrelation Matrix.

*Correlations*														
	Letter Sets	Numb. Ser.	Sci. Imp.: Creat.	Sci. Reas.: Generating Hyp.	Sci. Reas.: Generating Exp.	Sci. Reas.: Drawing Concl.	Sci. Imp.: Analytical	SAT Reading	SAT Math	GPA Undergrad	Research Experience	Lab Courses Taken	Research Meth. Course Taken	Numb. of Sci. Articles Read
Letter Sets	1.00	0.44 **	0.20	0.21	0.18	0.14	0.32 **	0.05	0.18	0.01	−0.35 **	−0.19	0.01	−0.01
Numb. Ser.	0.44 **	1.00	0.16	0.17	0.27 *	0.11	0.21	0.25*	0.31 *	0.08	−0.19	−0.20	0.09	−0.24 *
Sci. Imp.: Creat.	0.20	0.16	1.00	0.24 *	0.53 **	0.41 **	0.33 **	−0.05	0.01	0.20	0.14	−0.05	0.05	0.24 *
Sci. Reas.: Generating Hyp.	0.21	0.17	0.24 *	1.00	0.39 **	0.3 6 **	0.08	−0.13	0.06	0.10	0.16	0.11	0.16	−0.04
Sci. Reas.: Generating Exp.	0.18	0.27*	0.53 **	0.39 **	1.00	0.51 **	0.31 **	0.01	0.02	0.23	0.12	−0.03	0.08	0.24 *
Sci. Reas.: Drawing Concl.	0.14	0.11	0.41 **	0.36 **	0.51 **	1.00	0.18	0.03	0.22	0.18	0.36 **	0.20	0.31 **	0.31 **
Sci. Imp.: Analytical	0.32 **	0.21	0.33 **	0.08	0.31 **	0.18	1.00	0.16	−0.18	0.07	−0.02	0.00	−0.12	0.27 *
SAT Reading	0.05	0.25 *	−0.05	−0.13	0.01	0.03	0.16	1.00	0.32 **	0.08	−0.12	−0.10	0.00	0.11
SAT Math	0.18	0.31 *	0.01	0.06	0.02	0.22	−0.18	0.32 **	1.00	0.24	−0.06	0.02	0.05	0.05
GPA Undergrad	0.01	0.08	0.20	0.10	0.23	0.18	0.07	0.08	0.24	1.00	0.06	0.13	0.11	0.24 *
Research Experience	−0.35 **	−0.19	0.14	0.16	0.12	0.36 **	−0.02	−0.12	−0.06	0.06	1.00	0.17	0.26 *	0.26 *
Lab Courses Taken	−0.19	−0.20	−0.05	0.11	−0.03	0.20	0.00	−0.10	0.02	0.13	0.17	1.00	0.25 *	0.13
Research Meth. Course Taken	0.01	0.09	0.05	0.16	0.08	0.31 **	−0.12	0.00	0.05	0.11	0.26 *	0.25 *	1.00	0.05
Numb. Of Scic. Articles Read	−0.01	−0.24 *	0.24 *	−0.04	0.24 *	0.31 **	0.27 *	0.11	0.05	0.24 *	0.26 *	0.13	0.05	1.00

* *p* < 0.05. ** *p* < 0.01. All tests are two-tailed. Note: Correlations in the table are presented with pairwise deletion.

## Appendix B

Complete Set of Experimental Materials

You will be presented with items that each contain five sets of four letters. Four of those letter sets are similar to each other, and one is not. You will be asked to find a rule to determine what makes four of the sets alike and indicate which letter set does not fit the rule. Please cross out the letter set that does not fit.

Note:

Please note that the rules are not based on the sounds of letters, sounds of the letter sets, shapes of the letters, or whether combinations of those letters form words.

1. CDEF HIJK QRST IJKM OPQR
2. KLOP HOMT PLIS MORW OLSP

In example 1, four letter sets have letters in alphabetical order; one of them does not have letters in alphabetical order. Therefore, the letter set that does not have letters in alphabetical order was highlighted.

In example 2, four letter sets contain the letter O; one does not. Therefore, the letter set that does not contain the letter O was highlighted.

You will have 7 min to work on the items. You will not be able to move on to the next page until the 7 min are up so please take the full time to work on the items.

1.	CDEF	PQRS	JKLN	FGHI	MNOP
2.	GGHI	AABC	EERT	YTUW	DDJH
3.	TSST	VWWV	GHHG	ABBA	EFFE
4.	GHFA	YUTA	BHDA	NHJE	KLOA
5.	STUV	WXYZ	DEFG	GHIJ	LNMO
6.	HBJG	RTSR	AEIO	LKWN	VBCX
7.	TVYV	VHVJ	IHVV	WVYV	VQVT
8.	EETT	QQBB	JJKK	GGUU	PPWW
9.	RTVY	JKMO	EQVB	ICBX	LGHT
10.	YUTO	OVGF	ROTW	NWDC	QRSO
11.	CBFF	SDCG	EDEB	QQTV	TGHT
12.	SNOP	RSTU	BCDE	TUVW	HIJK
13.	RRRT	JUJJ	LLLE	TXXT	WAAA
14.	XEAT	XOUT	XYIT	XEOT	WLJT
15.	QGMO	LTUV	OXYZ	DENO	KLMN

Next, you will be presented with items that contain a series of numbers. You will be asked to find the number that should appear next in the series. Please write the number in the space provided at the end of each number series.

Let’s have a look at an example:

1. 2, 4, 6, 8, ______
2. 8, 8, 16, 16, 24, _____

In example 1, you derive each number by adding 2 to the previous one. Thus, the correct solution is 10.

In example 2, each number is repeated once. Thus, the correct solution is 24.

You will have 7 min to work on the items. You will not be able to move on to the next page until the 7 min are up so please take the full time to work on the items.

1. 6, 8, 12, 18, 26,____
2. 2, 7, 3, 8, 4, 9,____
3. 70, 70, 35, 35, 17.5,____
4. 42, 13, 44, 13, 46, 13,____
5. 24, 48, 62, 124, 81,____
6. 135, 45, 15,____
7. 67, 62, 60, 55, 53,____
8. 897, 880, 863, 846____
9. 3, 3, 3, 5, 5, 5, _____
10. 58, 31, 51, 40, 44, 49,____
11. 2, 3, 5, 7, 8, 15, 11, 3,____
12. 169, 144, 121, 100,____
13. 11, 13, 17, 23, 25, ____
14. 25, 5, 36, 6, 49____
15. 256, 16, 4, _____
16. 24, 24, 48, 72, 120____

In scientific research, some publications have more importance to society than others. These others are usually less applicable to current scientific or societal issues. Studies that are more impactful on society sometimes are designated as “high- impact” studies. Impact can be measured via the number of citations of a given published work. Scientific works that are cited very few times tend to have a lower impact on science and society. These works can be considered “low impact.” Using these definitions and understandings of “impact” in science, please complete the following tasks to the best of your ability.

**Task 1:**

Imagine you were given a task to design a high-impact study that would help understand the high rates of depression among adolescents. Your response should be approximately 200–300 words describing:

(a) the idea for the study, (b) the goal of the study, (c) your hypothesis or hypotheses regarding the outcomes of the study, (d) what you will be measuring, and (e) how the study would be performed.

**Task 2:**

In the context of the COVID-19 pandemic, design a high-impact study, as defined above, that you believe would be important in regard to the use of face masks. Your response should be approximately 200–300 words describing:

(a) the idea for the study, (b) the goal of the study, (c) your hypothesis or hypotheses regarding the outcomes of the study, (d) what you will be measuring, and (e) how the study would be performed.

**Task 3:**

Given the current climate crisis the world is facing today, formulate a high-impact study you would perform to express to current policymakers the urgent need for a policy change. Examples of issues regarding climate include: carbon emissions, pollution, overfishing, fertilizer run-off, etc... Your response should be approximately 200–300 words describing:

(a) the idea for the study, (b) the goal of the study, (c) your hypothesis or hypotheses regarding the outcomes of the study, (d) what you will be measuring, and (e) how the study would be performed.

In psychological science, some studies have high impact and are cited many times. Other studies have low impact and are hardly cited at all. We are seeking to determine whether students, after reading titles of studies, can determine whether particular studies are high-impact or low-impact. For each of the following studies, we would like to ask you six questions.

(1) Do you believe this study to be high-impact—cited many times—or low-impact— cited very few times?

If you believe the study to be high impact, write an “H.”

If you have the study to be low impact, write an “L.”

There are 10 high impact titles and 10 low impact. At the end, you may want to count how many times you put “H” and “L”. It should be 10 times for each.

(2) How confident are you in your rating?

If you have **high confidence** in your rating, write a “**3.**”

If you have **medium confidence** in your rating, write a “**2.**” If you have **low confidence** in your rating, write a “**1.**”

For the three following questions, please rate your answer on a scale of 1 to 3, as you did for the previous question. For example, for “how creative do you believe this work to be?”, if you believe the work to be **highly creative**, write a “**3**”, if you believe this to be **somewhat creative** work, write a “**2**,” and if you believe this to be only **slightly creative** work, write a “**1.**

(3) How creative do you believe this work to be?

3 = highly creative, 2 = somewhat creative, 1 = slightly creative

(4) How scientifically rigorous do you believe this work to be?

3 = highly rigorous, 2 = somewhat rigorous, 1 = slightly rigorous

(5) How practically useful do you believe this work to be in day-to-day life?

3 = highly practically useful, 2 = somewhat practically useful, 1 = slightly practically useful

On the next several pages, you will find various titles from papers that have been highly cited or have been rarely cited. They are in no particular order. Please answer the questions accordingly.

(1) An investigation of pesticide transport in soil and groundwater in the most vulnerable site of Bangladesh

Do you believe this study to be high-impact—cited many times—or low-impact— cited very few times (H or L)?

_____________

How confident are you in your rating (3 (high confidence), 2 (medium confidence), or 1 (low confidence))?

_____________

How creative do you believe this work to be (3 (highly creative), 2 (somewhat creative), 1 (slightly creative))?

_____________

How scientifically rigorous do you believe this work to be (3 (highly rigorous), 2 (somewhat rigorous), 1 (slightly rigorous))?

_____________

How practically useful do you believe this work to be (3 (highly practically useful), 2 (somewhat practically useful), 1 (slightly practically useful))?

_____________

(2) Will you leave me too?: The impact of father absence on the treatment of a 10-year- old girl

Do you believe this study to be high-impact—cited many times—or low-impact— cited very few times (H or L)?

_____________

How confident are you in your rating (3 (high confidence), 2 (medium confidence), or 1 (low confidence))?

_____________

How creative do you believe this work to be (3 (highly creative), 2 (somewhat creative), 1 (slightly creative))?

_____________

How scientifically rigorous do you believe this work to be (3 (highly rigorous), 2 (somewhat rigorous), 1 (slightly rigorous))?

_____________

How practically useful do you believe this work to be (3 (highly practically useful), 2 (somewhat practically useful), 1 (slightly practically useful))?

_____________

(3) Investigation of quality of sexual life and its impact factors in young female patients who have had a hysterectomy

Do you believe this study to be high-impact—cited many times—or low-impact— cited very few times (H or L)?

_____________

How confident are you in your rating (3 (high confidence), 2 (medium confidence), or 1 (low confidence))?

_____________

How creative do you believe this work to be (3 (highly creative), 2 (somewhat creative), 1 (slightly creative))?

_____________

How scientifically rigorous do you believe this work to be (3 (highly rigorous), 2 (somewhat rigorous), 1 (slightly rigorous))?

_____________

How practically useful do you believe this work to be (3 (highly practically useful), 2 (somewhat practically useful), 1 (slightly practically useful))?

_____________

(4) The “what” and “why” of goal pursuits: Human needs and the self-determination of behavior

Do you believe this study to be high-impact—cited many times—or low-impact— cited very few times (H or L)?

_____________

How confident are you in your rating (3 (high confidence), 2 (medium confidence), or 1 (low confidence))?

_____________

How creative do you believe this work to be (3 (highly creative), 2 (somewhat creative), 1 (slightly creative))?

_____________

How scientifically rigorous do you believe this work to be (3 (highly rigorous), 2 (somewhat rigorous), 1 (slightly rigorous))?

_____________

How practically useful do you believe this work to be (3 (highly practically useful), 2 (somewhat practically useful), 1 (slightly practically useful))?

_____________

(5) Evaluation of diets of young people aged 13–15 from rural areas in Karpatian Province in terms of diet-related disease risk in adulthood

Do you believe this study to be high-impact—cited many times—or low-impact— cited very few times (H or L)?

_____________

How confident are you in your rating (3 (high confidence), 2 (medium confidence), or 1 (low confidence))?

_____________

How creative do you believe this work to be (3 (highly creative), 2 (somewhat creative), 1 (slightly creative))?

_____________

How scientifically rigorous do you believe this work to be (3 (highly rigorous), 2 (somewhat rigorous), 1 (slightly rigorous))?

_____________

How practically useful do you believe this work to be (3 (highly practically useful), 2 (somewhat practically useful), 1 (slightly practically useful))?

_____________

(6) Effect of physical inactivity on major non-communicable diseases worldwide: An analysis of burden of disease and life expectancy

Do you believe this study to be high-impact—cited many times—or low-impact— cited very few times (H or L)?

_____________

How confident are you in your rating (3 (high confidence), 2 (medium confidence), or 1 (low confidence))?

_____________

How creative do you believe this work to be (3 (highly creative), 2 (somewhat creative), 1 (slightly creative))?

_____________

How scientifically rigorous do you believe this work to be (3 (highly rigorous), 2 (somewhat rigorous), 1 (slightly rigorous))?

_____________

How practically useful do you believe this work to be (3 (highly practically useful), 2 (somewhat practically useful), 1 (slightly practically useful))?

_____________

(7) Heart disease and stroke statistics-2012 update: A report from the American heart association

Do you believe this study to be high-impact—cited many times—or low-impact— cited very few times (H or L)?

_____________

How confident are you in your rating (3 (high confidence), 2 (medium confidence), or 1 (low confidence))?

_____________

How creative do you believe this work to be (3 (highly creative), 2 (somewhat creative), 1 (slightly creative))?

_____________

How scientifically rigorous do you believe this work to be (3 (highly rigorous), 2 (somewhat rigorous), 1 (slightly rigorous))?

_____________

How practically useful do you believe this work to be (3 (highly practically useful), 2 (somewhat practically useful), 1 (slightly practically useful))?

_____________

(8) Unequal Treatment: Confronting Racial and Ethnic Disparities in Health Care.

Do you believe this study to be high-impact—cited many times—or low-impact— cited very few times (H or L)?

_____________

How confident are you in your rating (3 (high confidence), 2 (medium confidence), or 1 (low confidence))?

_____________

How creative do you believe this work to be (3 (highly creative), 2 (somewhat creative), 1 (slightly creative))?

_____________

How scientifically rigorous do you believe this work to be (3 (highly rigorous), 2 (somewhat rigorous), 1 (slightly rigorous))?

_____________

How practically useful do you believe this work to be (3 (highly practically useful), 2 (somewhat practically useful), 1 (slightly practically useful))?

_____________

(9) Constructions of masculinity and their influence on men’s well-being: A theory of gender and health

Do you believe this study to be high-impact—cited many times—or low-impact— cited very few times (H or L)?

_____________

How confident are you in your rating (3 (high confidence), 2 (medium confidence), or 1 (low confidence))?

_____________

How creative do you believe this work to be (3 (highly creative), 2 (somewhat creative), 1 (slightly creative))?

_____________

How scientifically rigorous do you believe this work to be (3 (highly rigorous), 2 (somewhat rigorous), 1 (slightly rigorous))?

_____________

How practically useful do you believe this work to be (3 (highly practically useful), 2 (somewhat practically useful), 1 (slightly practically useful))?

_____________

(10) The positive effects of physical training on life quality in a 92-year-old female patient with exacerbation of chronic heart failure

Do you believe this study to be high-impact—cited many times—or low-impact— cited very few times (H or L)?

_____________

How confident are you in your rating (3 (high confidence), 2 (medium confidence), or 1 (low confidence))?

_____________

How creative do you believe this work to be (3 (highly creative), 2 (somewhat creative), 1 (slightly creative))?

_____________

How scientifically rigorous do you believe this work to be (3 (highly rigorous), 2 (somewhat rigorous), 1 (slightly rigorous))?

_____________

How practically useful do you believe this work to be (3 (highly practically useful), 2 (somewhat practically useful), 1 (slightly practically useful))?

_____________

(11) Relationship of childhood abuse and household dysfunction to many of the leading causes of death in adults: The adverse childhood experiences (ACE) study

Do you believe this study to be high-impact—cited many times—or low-impact— cited very few times (H or L)?

_____________

How confident are you in your rating (3 (high confidence), 2 (medium confidence), or 1 (low confidence))?

_____________

How creative do you believe this work to be (3 (highly creative), 2 (somewhat creative), 1 (slightly creative))?

_____________

How scientifically rigorous do you believe this work to be (3 (highly rigorous), 2 (somewhat rigorous), 1 (slightly rigorous))?

_____________

How practically useful do you believe this work to be (3 (highly practically useful), 2 (somewhat practically useful), 1 (slightly practically useful))?

_____________

(12) Nursing care experiences of a borderline personality patient with spiritual distress

Do you believe this study to be high-impact—cited many times—or low-impact— cited very few times (H or L)?

_____________

How confident are you in your rating (3 (high confidence), 2 (medium confidence), or 1 (low confidence))?

_____________

How creative do you believe this work to be (3 (highly creative), 2 (somewhat creative), 1 (slightly creative))?

_____________

How scientifically rigorous do you believe this work to be (3 (highly rigorous), 2 (somewhat rigorous), 1 (slightly rigorous))?

_____________

How practically useful do you believe this work to be (3 (highly practically useful), 2 (somewhat practically useful), 1 (slightly practically useful))?

_____________

(13) Evaluation of the environmental performance and rationing of water consumption in industrial production of beverages

Do you believe this study to be high-impact—cited many times—or low-impact— cited very few times (H or L)?

_____________

How confident are you in your rating (3 (high confidence), 2 (medium confidence), or 1 (low confidence))?

_____________

How creative do you believe this work to be (3 (highly creative), 2 (somewhat creative), 1 (slightly creative))?

_____________

How scientifically rigorous do you believe this work to be (3 (highly rigorous), 2 (somewhat rigorous), 1 (slightly rigorous))?

_____________

How practically useful do you believe this work to be (3 (highly practically useful), 2 (somewhat practically useful), 1 (slightly practically useful))?

_____________

(14) Seventh report of the Joint National Committee on Prevention, Detection, Evaluation, and Treatment of High Blood Pressure

Do you believe this study to be high-impact—cited many times—or low-impact— cited very few times (H or L)?

_____________

How confident are you in your rating (3 (high confidence), 2 (medium confidence), or 1 (low confidence))?

_____________

How creative do you believe this work to be (3 (highly creative), 2 (somewhat creative), 1 (slightly creative))?

_____________

How scientifically rigorous do you believe this work to be (3 (highly rigorous), 2 (somewhat rigorous), 1 (slightly rigorous))?

_____________

How practically useful do you believe this work to be (3 (highly practically useful), 2 (somewhat practically useful), 1 (slightly practically useful))?

_____________

(15) Culture, illness, and care. Clinical lessons from anthropologic and cross-cultural research

Do you believe this study to be high-impact—cited many times—or low-impact— cited very few times (H or L)?

_____________

How confident are you in your rating (3 (high confidence), 2 (medium confidence), or 1 (low confidence))?

_____________

How creative do you believe this work to be (3 (highly creative), 2 (somewhat creative), 1 (slightly creative))?

_____________

How scientifically rigorous do you believe this work to be (3 (highly rigorous), 2 (somewhat rigorous), 1 (slightly rigorous))?

_____________

How practically useful do you believe this work to be (3 (highly practically useful), 2 (somewhat practically useful), 1 (slightly practically useful))?

_____________

(16) Application of natural fermentation to ferment mulberry juice into alcoholic beverage

Do you believe this study to be high-impact—cited many times—or low-impact— cited very few times (H or L)?

_____________

How confident are you in your rating (3 (high confidence), 2 (medium confidence), or 1 (low confidence))?

_____________

How creative do you believe this work to be (3 (highly creative), 2 (somewhat creative), 1 (slightly creative))?

_____________

How scientifically rigorous do you believe this work to be (3 (highly rigorous), 2 (somewhat rigorous), 1 (slightly rigorous))?

_____________

How practically useful do you believe this work to be (3 (highly practically useful), 2 (somewhat practically useful), 1 (slightly practically useful))?

_____________

(17) The validity of the Hospital Anxiety and Depression Scale: An updated literature review

Do you believe this study to be high-impact—cited many times—or low-impact— cited very few times (H or L)?

_____________

How confident are you in your rating (3 (high confidence), 2 (medium confidence), or 1 (low confidence))?

_____________

How creative do you believe this work to be (3 (highly creative), 2 (somewhat creative), 1 (slightly creative))?

_____________

How scientifically rigorous do you believe this work to be (3 (highly rigorous), 2 (somewhat rigorous), 1 (slightly rigorous))?

_____________

How practically useful do you believe this work to be (3 (highly practically useful), 2 (somewhat practically useful), 1 (slightly practically useful))?

_____________

(18) Doctors on television: Analysis of doctors’ experiences during filming of a documentary in the workplace

Do you believe this study to be high-impact—cited many times—or low-impact— cited very few times (H or L)?

_____________

How confident are you in your rating (3 (high confidence), 2 (medium confidence), or 1 (low confidence))?

_____________

How creative do you believe this work to be (3 (highly creative), 2 (somewhat creative), 1 (slightly creative))?

_____________

How scientifically rigorous do you believe this work to be (3 (highly rigorous), 2 (somewhat rigorous), 1 (slightly rigorous))?

_____________

How practically useful do you believe this work to be (3 (highly practically useful), 2 (somewhat practically useful), 1 (slightly practically useful))?

_____________

(19) Decision-making in the physician-patient encounter: Revisiting the shared treatment decision-making model

Do you believe this study to be high-impact—cited many times—or low-impact— cited very few times (H or L)?

_____________

How confident are you in your rating (3 (high confidence), 2 (medium confidence), or 1 (low confidence))?

_____________

How creative do you believe this work to be (3 (highly creative), 2 (somewhat creative), 1 (slightly creative))?

_____________

How scientifically rigorous do you believe this work to be (3 (highly rigorous), 2 (somewhat rigorous), 1 (slightly rigorous))?

_____________

How practically useful do you believe this work to be (3 (highly practically useful), 2 (somewhat practically useful), 1 (slightly practically useful))?

_____________

(20) A role for community health of a traditional birth attendant working in a Nicaraguan rural area

Do you believe this study to be high-impact—cited many times—or low-impact— cited very few times (H or L)?

_____________

How confident are you in your rating (3 (high confidence), 2 (medium confidence), or 1 (low confidence))?

_____________

How creative do you believe this work to be (3 (highly creative), 2 (somewhat creative), 1 (slightly creative))?

_____________

How scientifically rigorous do you believe this work to be (3 (highly rigorous), 2 (somewhat rigorous), 1 (slightly rigorous))?

_____________

How practically useful do you believe this work to be (3 (highly practically useful), 2 (somewhat practically useful), 1 (slightly practically useful))?

_____________

Answer Key:

1. Low
2. Low
3. Low
4. High
5. Low
6. High
7. High
8. High
9. High
10. Low
11. High
12. Low
13. Low
14. High
15. High
16. Low
17. High
18. Low
19. High
20. Low

On the next page, you will read several scenarios that describe a situation as well as a hypothesis to explain the facts presented. You will be asked to think of some other explanations (alternative hypotheses) that can explain the circumstances presented. Please write down any alternative explanations you can think of. There is no time limit for this exercise.

Example: Eve is interested in studying the effects of taking exams on student performance. She devises an experiment where group A students are given weekly quizzes and bi-semester exams, while group B students are only given bi-semester exams. The results show that students in group A do better overall than do students in group B. She explains that weekly quizzes help the students stay on track with the material.

*What are some alternative hypotheses regarding why the students who receive weekly quizzes perform better than the students who don’t?*

Potential answers:

- It may be that the quizzes allow the students in group A to make mistakes and learn from them before taking the exams.
- It may be that the students in group A are simply better exam takers than students in group B, regardless of any prior exams they may have taken.
- It may be that students in group B are not used to the types of questions that Eve tends to ask on the quizzes/exams.
- It may be that there are more students who are good at math in group A than group B, which skews the data.
- It may be that Group A was exposed to other variables (e.g., a more effective teacher) which would have resulted in higher scores than group B, even if they were not taking weekly exams.
- It may be that the exams are biased towards students in Group A, say by asking the same questions that were already assessed in the quizzes.

1. Marie is interested in child development. One day, she notices that whenever Laura’s nanny comes in to pick up Laura from nursery school, Laura starts to cry. Marie reflects upon how sad it is that Laura has a poor relationship with her nanny.

*What are some alternative hypotheses regarding why Laura starts to cry when she is picked up from nursery school by the nanny?*

2. Jane is interested in the relationship between HIV/AIDS illness and depression. In one study, she finds that 10% of subjects without HIV/AIDS are clinically depressed, whereas 60% of subjects with HIV/AIDS are clinically depressed. Upon consideration of the data, Jane hypothesizes that subjects with HIV/AIDS are more likely to develop clinical depression because they are aware of their critical condition and often feel hopeless about it.

*What are some alternative hypotheses regarding why subjects with HIV/AIDS are more likely to develop clinical depression?*

On the next page, you will read several scenarios that describe a situation as well as a hypothesis. You will be asked to design an experiment for each of those scenarios to test the hypothesis presented. There is no time limit for this exercise.

Note:

You do not need to be familiar with specific tests in any of the subject areas presented. For example, if you want to assess stereotypes toward a target group and are not familiar with tests that assess stereotypes, just write in your answer that a test assessing stereotypes toward a particular target group should be administered.

Here is an example:

Martin believes that a particular yellow food dye (E104) not only causes hyperactivity in children (as has been proven), but also increases people’s divergent thinking. That is, he believes this dye puts people in a state in which they are more creative. How can he test his hypothesis that the dye E104 increases creativity?

Possible answer:

Recruit 100 participants. Give half of them a beverage that contains E104, and half of them a similar beverage without that dye. After half an hour, assess their creativity by means of several tests like the Torrance test of divergent thinking, the alternative uses test by Guilford, or the cartoon caption test by Sternberg

1. Ella, a senior in college, observes that her roommate tends to perform better on an exam if she has had a cup of coffee beforehand. Ella hypothesizes that drinking coffee before taking an exam will significantly increase one’s exam performance. However, Ella does not know how to test this hypothesis.

*Please suggest an experimental design to test this hypothesis and describe the experiment in some detail. Assume you have the resources you need to be able to do the experiment (e.g., access to students and their academic records, sufficient funds to pay subjects, etc.).*

2. John hypothesizes that his brother’s playing of violent video games has increased his brother’s aggressive behavior. John is not sure, however, whether playing violent video games really increases aggression.

*Please suggest an experimental design to test John’s hypothesis and describe the experiment in some detail. Assume you have the resources you need to be able to do the experiment (e.g., access to violent video games, subjects, sufficient funds to pay subjects, etc.).*

On the next page, you will read several scenarios that describe an experiment that was conducted to test a specific hypothesis. However, each one of these experiments is flawed in some way. You will be asked to consider the experimental design and point out the flaws. Please write down any flaws you can come up with (one is enough but if you can think of more, then please write them down as well). There is no time limit for this exercise.

Example:

We tested the hypothesis that when a salesperson smiles directly at a customer, the individual is more likely to make a sale than when the salesperson fails to smile. Five saleswomen at a bridal shop were instructed to do one of three things while trying to sell a wedding dress to a customer: either to smile directly (in the face of) the customer, smile indirectly (while looking away from) the customer, or have a neutral expression on the face. It was found that smiling directly at customers did indeed increase sales significantly. Fewest wedding dresses were bought in the indirect-smiling condition. It was concluded that salespeople should smile directly into the faces of their customers if they wish to increase their sales effectiveness.

*Is this conclusion correct? Why or why not?*

Possible Answer:

The conclusion is not correct because:

1. All customers were women so one cannot generalize to all customers.
2. All salespeople were women so one cannot generalize to all salespeople.
3. Bridal-shop customers are not representative of customers in general.
4. The conclusion that looking away from customers (indirect smiling condition) was crucial in producing the lowest sales would not follow conclusively unless there were two clear conditions in which the salesperson had a neutral expression, either looking directly at the customer or looking away from the customer

1. Bill was interested in how well a new program for improving mathematical performance worked. He gave 200 students a pretest on their mathematical knowledge and skills. He then administered the new program to them. After administering the program, he gave the same 200 students a posttest that was equal in difficulty and in all relevant ways comparable to the pretest. He found that students improved significantly in performance from pretest to posttest. He concluded that the program for improving mathematical performance was effective.

*Is this conclusion correct? Why or why not?*

2. Mary believed that administering 400 milligrams of Vitamin C two hours before a test would improve performance on the test. She randomly assigned 200 subjects either to Group A or Group B. She administered 400 milligrams of Vitamin C to Group A subjects and then gave them a test two hours later. She also had a control group, Group B, to which she administered nothing at all. She just gave them the same test Group A had gotten. She found that Group A performed at a higher level than did Group B. She concluded that 400 milligrams of Vitamin C administered two hours before the test improved subjects’ performance.

*Is this conclusion correct? Why or why not?*

**Questionnaire**

Please fill out the responses below. Please be honest with your answers. Keep in mind we are collecting these data anonymously.

What is your gender? Male ___ Female_____ Other______

How old are you? ______________ years

What year are you in at Cornell? ______________

What is your major? __________________________

What is your Cornell cumulative GPA to date? ______________

What was your highest critical reading SAT score? ______________

What was your highest math SAT score? ______________

What was your highest ACT reading score? _________________

What was your highest ACT math score? __________________

If you have taken the GRE, what was your score? _____________

Do you have any experience conducting studies or experiments? If so, what did you do?

Number of lab courses you have taken _____________

Have you taken a class on research methods? Yes No

How many scientific articles do you read per month? ___________

What is your ethnicity?

African or African-American ________

Asian or Asian-American ________

European or European-American ________

Hispanic or Hispanic-American ________

Other ________

No response _______

## References

[B1-jintelligence-13-00129] ACT, and College Board (2018). ACT/SAT Concordance Tables. https://www.act.org/content/dam/act/unsecured/documents/ACT-SAT-Concordance-Tables.pdf.

[B2-jintelligence-13-00129] Bernstein Brian O., Lubinski David, Benbow Camilla P. (2019). Psychological constellations assessed at age 13 predict distinct forms of eminence 35 years later. Psychological Science.

[B3-jintelligence-13-00129] Carroll John B. (1993). Human Cognitive Abilities: A Survey of Factor-Analytic Studies.

[B4-jintelligence-13-00129] Cole James S., Gonyea Robert M. (2010). Accuracy of Self-Reported SAT and ACT Test Scores: Implications for Research. Research in Higher Education.

[B5-jintelligence-13-00129] Deary Ian J. (2020). Intelligence: A Very Short Introduction.

[B6-jintelligence-13-00129] Dougherty Michael R., Horne Zachary (2022). Citation counts and journal impact factors do not capture some indicators of research quality in the behavioural and brain sciences. Royal Society Open Science.

[B7-jintelligence-13-00129] Dunbar Kevin, Sternberg Robert J., Davidson Janet E. (1995). How scientists really reason: Scientific reasoning in real-world laboratories. The Nature of Insight.

[B8-jintelligence-13-00129] Dunbar Kevin N., Klahr David, Holyoak Keith J., Morrison Robert G. (2012). Scientific thinking and reasoning. The Oxford Handbook of Thinking and Reasoning.

[B9-jintelligence-13-00129] Feyerabend Paul (2010). Against Method.

[B10-jintelligence-13-00129] Frey Meredith C., Detterman Douglas K. (2004). Scholastic assessment or *g*? The relationship between the Scholastic Assessment Test and general cognitive ability. Psychological Science.

[B11-jintelligence-13-00129] Guyote Martin J., Sternberg Robert J. (1981). A transitive-chain theory of syllogistic reasoning. Cognitive Psychology.

[B12-jintelligence-13-00129] Hirsch Jorge E. (2005). An index to quantify an individual’s scientific research output. Proceedings of the National Academy of Sciences.

[B13-jintelligence-13-00129] Hirsch Jorge E. (2007). Does the *h* index have predictive power?. Proceedings of the National Academy of Sciences.

[B14-jintelligence-13-00129] Hirsch Jorge E. (2010). An index to quantify an individual’s scientific research output that takes into account the effect of multiple coauthorship. Scientometrics.

[B15-jintelligence-13-00129] Jensen Arthur R. (1998). The g Factor.

[B16-jintelligence-13-00129] Koenig Katherine A., Frey Meredith C., Detterman Douglas K. (2008). ACT and general cognitive ability. Intelligence.

[B17-jintelligence-13-00129] Koslowski Barbara (1996). Theory and Evidence: The Development of Scientific Reasoning.

[B19-jintelligence-13-00129] Kuhn Deanna, Goswami Usha (2002). What is scientific thinking, and how does it develop?. Blackwell Handbook of Childhood Cognitive Development.

[B20-jintelligence-13-00129] Kuhn Deanna, Goswami Usha (2011). What is scientific thinking and how does it develop?. The Wiley-Blackwell Handbook of Childhood Cognitive Development.

[B21-jintelligence-13-00129] Kuhn Deanna, Dean Chipper (2004). Connecting scientific reasoning and causal inference. Journal of Cognition and Development.

[B18-jintelligence-13-00129] Kuhn Thomas S. (1970). The Structure of Scientific Revolutions.

[B22-jintelligence-13-00129] Kuncel Nathan R., Hezlett Sarah A. (2007). Standardized tests predict graduate students’ success. Science.

[B23-jintelligence-13-00129] Kuncel Nathan R., Hezlett Sarah A., Ones Deniz S. (2001). A comprehensive meta-analysis of the predictive validity of the graduate record examinations: Implications for graduate student selection and performance. Psychological Bulletin.

[B24-jintelligence-13-00129] Kuncel Nathan R., Wee Serena, Serafin Lauren, Hezlett Sarah A. (2010). The validity of the Graduate Record Examination for master’s and doctoral programs: A meta-analytic investigation. Educational and Psychological Measurement.

[B25-jintelligence-13-00129] Margolis J. (1967). Citation indexing and evaluation of scientific papers. Science.

[B26-jintelligence-13-00129] McCabe Kira O., Lubinski David, Benbow Camilla P. (2020). Who shines most among the brightest?: A 25-year longitudinal study of elite STEM graduate students. Journal of Personality and Social Psychology.

[B27-jintelligence-13-00129] McGrew Kevin S., Flanagan Dawn P., Harrison Patti L. (2005). The Cattell-Horn-Carroll theory of cognitive abilities: Past, present, and future. Contemporary Intellectual Assessment: Theories, Tests, Issues.

[B28-jintelligence-13-00129] NCES (2017). Digest of Education Statistics. Table 226.30. Number, Percentage Distribution, and SAT Mean Scores of High School Seniors Taking the SAT, by High School Grade Point Average, Intended College Major, and Degree-Level Goal: 2017. https://nces.ed.gov/programs/digest/d17/tables/dt17_226.30.asp?.

[B29-jintelligence-13-00129] Nisbett Richard E. (2016). Mindware: Tools for Smart Thinking.

[B30-jintelligence-13-00129] Popper Karl (2014). The Logic of Scientific Discovery.

[B31-jintelligence-13-00129] Radicchi Filippo, Fortunato Santo, Castellano Claudio (2008). Universality of citation distributions: Toward an objective measure of scientific impact. Proceedings of the National Academy of Sciences.

[B32-jintelligence-13-00129] Sackett Paul R., Shewach Oren R., Dahlke Jeffrey A., Sternberg Robert J. (2020). The predictive value of general intelligence. Human Intelligence: An Introduction.

[B33-jintelligence-13-00129] Schneider Lisa M., Briel Jacqueline B. (1990). Validity of the GRE: 1989–1989 Summary Report.

[B34-jintelligence-13-00129] Spearman Charles (1927). The Abilities of Man.

[B35-jintelligence-13-00129] Sternberg Robert J. (1985). Beyond IQ: A Triarchic Theory of Human Intelligence.

[B36-jintelligence-13-00129] Sternberg Robert J. (1994). Encyclopedia of Human Intelligence.

[B37-jintelligence-13-00129] Sternberg Robert J. (1997). Successful Intelligence: A New Theory of Intelligence.

[B38-jintelligence-13-00129] Sternberg Robert J., Sternberg Robert J., Fiske Susan T., Foss Donald J. (2016). What makes a psychological scientist “eminent”?. Scientists Making a Difference: One Hundred Eminent Behavioral and Brain Scientists Talk About Their Most Important Contributions.

[B39-jintelligence-13-00129] Sternberg Robert J. (2019). A theory of adaptive intelligence and its relation to general intelligence. Journal of Intelligence.

[B40-jintelligence-13-00129] Sternberg Robert J. (2020). It’s Time to Stem Malpractice in STEM Admissions. Inside Higher Ed. https://www.insidehighered.com/views/2020/07/28/colleges-shouldnt-use-standardized-admissions-tests-alone-measure-scientific.

[B41-jintelligence-13-00129] Sternberg Robert J., Grigorenko Elena L. (2006). Cultural intelligence and successful intelligence. Group & Organization Management.

[B44-jintelligence-13-00129] Sternberg Robert J., Sternberg Karin (2017). Measuring scientific reasoning for graduate admissions in psychology and related disciplines. Journal of Intelligence.

[B42-jintelligence-13-00129] Sternberg Robert J., Spear-Swerling Louise (1999). Enseñar a Pensar.

[B43-jintelligence-13-00129] Sternberg Robert J., Gordeeva Tamara (1996). The anatomy of impact: What makes an article influential?. Psychological Science.

[B45-jintelligence-13-00129] Sternberg Robert J., Wong Chak H., Sternberg Karin (2019). The relation of tests of scientific reasoning to each other and to tests of fluid intelligence. Journal of Intelligence.

[B46-jintelligence-13-00129] Sternberg Robert J., Siriner Ilaria, Oh Jamie, Wong Chak H. (2022). Cultural intelligence: What is it and how can it effectively be measured?. Journal of Intelligence.

[B47-jintelligence-13-00129] Sternberg Robert J., Sternberg Karin, Todhunter Rebel J. E. (2017). Measuring reasoning about teaching for graduate admissions in psychology and related disciplines. Journal of Intelligence.

[B48-jintelligence-13-00129] Sternberg Robert J., Todhunter Rebel J., Litvak Aaron, Sternberg Karin (2020). The relation of scientific creativity and evaluation of scientific impact to scientific reasoning and general intelligence. Journal of Intelligence.

[B49-jintelligence-13-00129] U.S. Department of Education, National Center for Education Statistics (2023). Average SAT Scores and Standard Deviations by State. https://nces.ed.gov/programs/digest/d23/tables/dt23_226.40.asp.

[B50-jintelligence-13-00129] Wilson Kenneth M. (1979). The Validation of GRE Scores as Predictors of First Year Performance in Graduate Study: Report of the GRE Cooperative Validity Studies Project.

